# New generalized variable stepsizes of the CQ algorithm for solving the split feasibility problem

**DOI:** 10.1186/s13660-017-1409-9

**Published:** 2017-06-12

**Authors:** Peiyuan Wang, Jianjun Zhou, Risheng Wang, Jie Chen

**Affiliations:** 1 Postdoctoral workstation, China Marine Development and Reserch Center (CMDRC), P.O. Box 1301, Beijing, 102249 China; 2 Naval Aviation University, Yantai, 266041 China

**Keywords:** 46E20, 46H35, 47H14, 47L30, split feasibility problem, CQ algorithm, variable stepsize, generalized variable stepsize

## Abstract

Variable stepsize methods are effective for various modified CQ algorithms to solve the split feasibility problem (SFP). The purpose of this paper is first to introduce two new simpler variable stepsizes of the CQ algorithm. Then two new generalized variable stepsizes which can cover the former ones are also proposed in real Hilbert spaces. And then, two more general KM (Krasnosel’skii-Mann)-CQ algorithms are also presented. Several weak and strong convergence properties are established. Moreover, some numerical experiments have been taken to illustrate the performance of the proposed stepsizes and algorithms.

## Introduction

Since the CQ algorithm for solving the split feasibility problem (SFP) was proposed [1] in order to get better convergence speed, much attention has been paid to improve the variable stepsize of CQ algorithm.

Let $H_{1}$ and $H_{2}$ be real Hilbert spaces, *C* and *Q* be nonempty closed convex subsets of $H_{1}$ and $H_{2}$, respectively, and $A:H_{1} \to H_{2} $ be a bounded linear operator. In this setting, the SFP [2] is formulated as finding a point *x̂* with the requirement

1.1 $$ \hat{x} \in C \quad\text{and} \quad A\hat{x} \in Q. $$

Denote the set of solutions for the SFP by $\Gamma= C \cap A^{ - 1}(Q)$, and define $f:{H_{1}} \to R $ by

$$f(x) = \frac{1}{2}{ \bigl\Vert {(I - {P_{Q}})Ax} \bigr\Vert ^{2},} $$

we see the function $f(x)$ is convex and continuously differentiable.

In [1, 3], Byrne proposed his CQ algorithm; it generates a sequence $\{x_{n} \}$ via the recursion

1.2 $$ x_{n + 1} = P_{C} \bigl(I - \tau_{n} A^{\ast}(I - P_{Q} )Ax_{n} \bigr), \quad n \ge0, $$

where initial $x_{0} \in H_{1} $ and $\tau_{n} \in ( {0,2 / \Vert A \Vert }^{2} )$, $P_{C}$ and $P_{Q}$ are the orthogonal projections onto *C* and *Q*, respectively.

Considering that the projection onto the nonempty closed convex sets *C* and *Q* might be hard to be implemented, Yang [4] and Xu [5] showed that if *C* and *Q* are level sets of convex functions, it just needs to project onto half-spaces $C_{n}$ and $Q_{n}$, thus the so-called relaxed CQ algorithm is reduced to the following formula:

1.3 $$ x_{n + 1} = P_{C_{n} } \bigl(I - \tau_{n} A^{\ast}(I - P_{Q_{n} } )Ax_{n} \bigr), \quad n \ge0. $$

In (1.3), the projections $P_{C_{n} } $ and $P_{Q_{n} } $ have closed-form expressions, thus they are easily to be computed. Define $f_{n} :H_{1} \to \mathrm{R}$ by

$$f_{n} (x) = \frac{1}{2} \bigl\Vert {(I - P_{Q_{n} } )Ax} \bigr\Vert ^{2}, $$

then the convex objective $f_{n}$ is also differentiable and has a Lipschitz gradient given by

$$\nabla f_{n} (x) = A^{\ast}(I - P_{Q_{n} } )Ax. $$

Noting that when applying (1.2) and (1.3) to solve the practical problems such as signal processing and image reconstruction, which can be covered by the SFP, it is hard to avoid that a fixed stepsize related to the norm of *A* sometimes affects convergence of the algorithms. Therefore, in order not to compute the matrix inverse and the largest eigenvalue of the matrix $A^{T}A$ and have a sufficient decrease of the objective function at each iteration, people have invented various algorithms with a variable or self-adaptive stepsize. Since Qu [6] presented a searching method by adopting Armijo-like search, many similar methods have been proposed, such as [7–12] etc. However, through these methods, self-adaptive stepsize at each iteration can be achieved, most formats of them are becoming more complex, it is difficult to apply them to some practical problems, and this needs considerable time complexity, especially for the large-scale setting and sparse problem.

On the other hand, another way to construct the variable stepsize without calculating matrix norm was proposed by Yang in [13], that is,

1.4 $$ \tau_{n} : = \frac{\rho_{n} }{ \Vert {\nabla f(x_{n} )} \Vert }, $$

where $\{ {\rho_{n} } \}$ is a sequence of positive real numbers such that

$$\sum_{n = 0}^{\infty}{\rho_{n} = \infty} , \qquad \sum_{n = 0}^{\infty}{ \rho_{n}^{2} < \infty} . $$

at the same time, the additional conditions that *Q* is a bounded subset and *A* is a full column rank matrix are required. Wang *et al*. [14] applied (1.4) to solve the SFP. Afterwards, in order to remove the two additional conditions, López *et al*. [15] introduced another choice of the stepsize sequence $\{ {\tau_{n} } \}$ as follows:

1.5 $$ \tau_{n} : = \frac{\rho_{n} f_{n} (x_{n} )}{ \Vert {\nabla f_{n} (x_{n} )} \Vert ^{2}}, $$

where $\{ {\rho_{n} } \}$ is chosen in the interval $(0,4)$. Furthermore, in paper [16] we were aware that if $\nabla f_{n} (x_{n} ) = 0$ in (1.5) for some $n \ge1$, the corresponding algorithms in [15] have to be terminated. In this case, $x_{n} $ may not be in *C* and is not necessarily a solution of SFP (1.1). With this observation, we have introduced a stepsize sequence $\{ {\tau_{n} } \}$ as follows:

1.6 $$ \tau_{n} : = \frac{\rho_{n} f_{n} (x_{n} )}{ ( { \Vert {\nabla f_{n} (x_{n} )} \Vert + \omega_{n} } )^{2}}, $$

where $\{ {\rho_{n} } \}$ is also chosen in the interval $(0,4)$ and $\{ {\omega_{n} } \}$ is a sequence of positive numbers in $(0,1)$. This choice of the stepsize sequence makes the associated algorithms never terminate unless the solution of SFP has been found. However, there exists inconvenience dealing with the choice of the parameter $\omega_{n} $. Only when it is a small number, a similar convergence speed as adopting (1.5) can be guaranteed. After observing many experiments, the order of magnitude for $\omega_{n} $ usually about less than 10^−5^ can satisfy that, it closely relates with the bit of computer and the float precision of the calculation software.

In order to improve these and avoid the calculations of $f_{n} (x)$ or $\nabla f_{n} (x_{n} )$, in this paper, we firstly propose two simpler choices of a stepsize as follows:

1.7 $$ \tau_{n} : = \frac{\rho_{n} \Vert {x_{n} - \bar{x}_{n} } \Vert ^{2}}{ \Vert {Ax_{n} - A\bar{x}_{n} } \Vert ^{2}} $$

and

1.8 $$ \tau_{n} : = \frac{\rho_{n} \Vert {\bar{x}_{n} } \Vert ^{2}}{ \Vert {A\bar {x}_{n} } \Vert ^{2}}, $$

where $\{ {\rho_{n} } \}$ is chosen in the interval $(0,2)$, $x_{n} \ne\bar{x}_{n} $, and $\bar{x}_{n} \ne0$. The advantages of our choices (1.7) and (1.8) lie in facts that they not only possess simpler formats easily to be calculated and implemented in practice, but also have significantly faster convergence speed, especially in a large-scale setting and sparse problem. Secondly, we present two generalized variable stepsizes as follows:

1.9 $$ \tau_{n} : = \frac{\rho_{n} \Vert {r(x_{n} )} \Vert ^{2}}{ \Vert {Ar(x_{n} )} \Vert ^{2}} $$

or

1.10 $$ {\tau_{n}}: = \frac{{{\rho_{n}}{{ \Vert {p({x_{n}})} \Vert }^{2}}}}{{{{ \Vert {{A^{T}}p({x_{n}})} \Vert }^{2}}}}, $$

where $\{ {\rho_{n} } \}$ is chosen in the interval $(0,2)$, and $r(x_{n})\ne0$ and $p(x_{n})\ne0$. Consequently, stepsizes (1.5)-(1.8) are the special cases of (1.9) or (1.10), and many similar stepsize formats can be obtained from them.

Recently, Yao *et al*. [17] have applied (1.5) to an improved CQ algorithm with a generalized Halpern iteration. In paper [18], we have modified the relative parameters with satisfactory conditions. Then, in this paper, we combine the iterations in [17, 18] with the KM-CQ iterations in [19, 20]. We propose two more general KM-CQ algorithms with the generalized variable stepsize (1.9) or (1.10), and they can be used to approach the minimum norm solution of the SFP that solves special variational inequalities.

The rest of this paper is organized as follows. Section 2 reviews some propositions and known lemmas. Section 3 gives two modified CQ algorithms with simpler variable stepsizes and shows the weak convergence. Section 4 presents two general KM-CQ algorithms with the generalized variable stepsizes and proves the strong convergence. In Section 5, we include numerical experiments to testify the better performance of the proposed stepsizes and algorithms with typical problems of signal processing and image restoration. Finally, Section 6 gives some conclusions and further research aim.

## Preliminaries

Let *H* be a real Hilbert space with the inner product $\langle{ \cdot, \cdot} \rangle$ and the norm $\Vert \cdot \Vert $, respectively. Let *C* be a nonempty closed convex subset of *H*. We will use the following notations:

- ‘→’ stands for strong convergence;
- ‘⇀’ stands for weak convergence;
- *I* stands for the identity mapping on *H*.

Recall that a mapping $T:C \to H$ is nonexpansive iff $\Vert {Tx - Ty} \Vert \le \Vert {x - y} \Vert $ for all $x,y \in C$.

A mapping $\psi:C \to H$ is said to be *δ*-contractive iff there exists a constant $\delta\in[0,1)$ such that

$$\bigl\Vert {\psi(x) - \psi(y)} \bigr\Vert \le\delta \Vert {x - y} \Vert $$

for all $x,y \in C$.

Recall also that the nearest point projection from *H* onto *C*, denoted by $P_{C} $, assigns to each $x \in H$ the unique point $P_{C} x \in C$ with the property $\Vert {x - P_{C} x} \Vert \le \Vert {x - y} \Vert $, $\forall y \in C$. We collect the basic properties of $P_{C} $ as follows.

### Proposition 2.1

[6, 21]

($\mathrm{p}_{1}$)
$\langle{x - P_{C} x,y - P_{C} x} \rangle \le0$ *for all* $x \in H$, $y \in C$;
($\mathrm{p}_{2}$)
$\Vert {P_{C} x - P_{C} y} \Vert ^{2} \le \langle{P_{C} x - P_{C} y,x - y} \rangle$ *for every* $x,y \in H$;
($\mathrm{p}_{3}$)
$\Vert {P_{C} x - P_{C} y} \Vert ^{2} \le \Vert {x - y} \Vert ^{2} - \Vert {(I - P_{C} )x - (I - P_{C} )y} \Vert ^{2}$ *for all* $x,y \in H$;
($\mathrm{p}_{4}$)
$\langle{(I - P_{C} )x - (I - P_{C} )y,x - y} \rangle\ge \Vert {(I - P_{C} )x - (I - P_{C} )y} \Vert ^{2}$ *for all* $x,y \in H$;
($\mathrm{p}_{5}$)
$\Vert {P_{C} x - z} \Vert ^{2} \le \Vert {x - z} \Vert ^{2} - \Vert {P_{C} x - x} \Vert ^{2}$ *for all* $x \in H$, $z\in C$.

In a Hilbert space *H*, the next following facts are well known.

### Proposition 2.2

$\forall x,y \in H$, $\forall t \in \mathrm{R}$,

(i) $\Vert {x\pm y} \Vert ^{2} = \Vert x \Vert ^{2}\pm2 \langle {x,y} \rangle+ \Vert y \Vert ^{2}$;
(ii) $\Vert {tx + (1 - t)y} \Vert ^{2} = t \Vert x \Vert ^{2} + (1 - t) \Vert y \Vert ^{2} - t(1 - t) \Vert {x - y} \Vert ^{2}$.

*For the SFP*, *we assume that the following conditions are satisfied in a Hilbert space* [5]:

(i) *The solution set of the SFP is nonempty*.

(ii) *The set* *C* *is given by*

$$C = \bigl\{ {x \in H_{1} | {c(x) \le0} } \bigr\} , $$

*where* $c:H_{1} \to\mathrm{R}$ *is a convex function and* *C* *is nonempty*.

*The set* *Q* *is given by*

$$Q = \bigl\{ {y \in H_{2} | {q(y) \le0} } \bigr\} , $$

*where* $q:H_{2} \to\mathrm{R}$ *is a convex function and* *Q* *is nonempty*.

(iii) *Assume that both* *c* *and* *q* *are subdifferentiable on* $H_{1} $ *and* $H_{2} $, *respectively*. *The subdifferentials are defined as follows*:

$$\partial c(x) = \bigl\{ {\xi\in H_{1} | {c(z) \ge c(x) + \langle { \xi,z - x} \rangle}\textit{ for all }z \in H_{1} } \bigr\} \ne \emptyset, $$

*for all* $x \in C$, *and*

$$\partial q(y) = \bigl\{ {\eta\in H_{2} | {q(u) \ge q(y) + \langle { \eta,u - y} \rangle}\textit{ for all }u \in H_{2} } \bigr\} \ne \emptyset, $$

*for all* $y \in Q$.

(iv) *We also assume that* *∂c* *and* *∂q* *are bounded on bounded sets*. *The set* $C_{n}$ *is given by*

$$C_{n} = \bigl\{ {x \in H_{1} | {c(x_{n} ) + \langle{\xi_{n} ,x - x_{n} } \rangle\le0} } \bigr\} , $$

*where* $\xi_{n} \in\partial c(x_{n} )$. *The set* $Q_{n}$ *is constructed by*

$$Q_{n} = \bigl\{ {y \in H_{2} | {q(Ax_{n} ) + \langle{\eta_{n} ,y - Ax_{n} } \rangle\le0} } \bigr\} , $$

*where* $\eta_{n} \in\partial q(Ax_{n} )$.

It is easily seen that $C \subseteq C_{n} $ and $Q \subseteq Q_{n} $ for all *n*.

The relaxed CQ algorithm [4] can be seen as a special case of the classical gradient projection method (GPM). To see this, we can consider the following convex minimization problem:

2.1 $$ \min_{x \in C_{n} } f_{n}(x). $$

It is well known that $\hat{x} \in C_{n} $ is a solution of problem (2.1) if and only if

2.2 $$ \bigl\langle {\nabla f_{n} (\hat{x}),x - \hat{x}} \bigr\rangle \ge0, \quad \forall x \in C_{n} . $$

Also, we know that (2.2) holds true if and only if

$$\hat{x} = P_{C_{n} } (I - \tau\nabla f_{n} )\hat{x}, \quad \forall\tau> 0. $$

Therefore, we use the GPM to solve the SFP, for any $x_{0} \in H_{1} $,

2.3 $$ x_{n + 1} = P_{C_{n} } \bigl[ {x_{n} - \tau_{n} \nabla f_{n }(x_{n} )} \bigr], \quad n \ge0, $$

where $\tau_{n} \in(0,2 / L)$, while *L* is the Lipschitz constant of $\nabla f_{n} $. Noting that $L = \Vert A \Vert ^{2}$, we see that (2.3) is exactly the relaxed CQ algorithm (1.3) .

### Proposition 2.3

see [22]

*Let* $\{\alpha_{n} \}$ *be a sequence of nonnegative real numbers such that*

$$\alpha_{n + 1} \le(1 - t_{n} )\alpha_{n} + t_{n} b_{n} , \quad n \ge1, $$

*where* $\{ {t_{n} } \}$ *is a sequence in* $(0,1)$ *and* $b_{n} \in\mathbb{R}$ *such that*

(i) $\sum_{n = 1}^{\infty}{t_{n} = \infty} $;
(ii) $\overline{\lim} _{n} b_{n} \le0$ *or* $\sum_{n = 1}^{\infty}{ \vert {t_{n} b_{n} } \vert } < \infty$.

*Then* $\alpha_{n} \to0$.

### Proposition 2.4

[23, 24]

*For some countable index set* Λ, *we denote by* $\ell _{p}=\ell_{p}(\Lambda)$, $1\le p\le\infty$. *Soft*-*thresholding leads to the unique minimizer of a functional combining* $\ell _{2}$ *and* $l_{1}$-*norms*,

2.4 $$ \mathrm{S}_{\mu}(a) = \arg\min_{x \in\ell_{2} (\Lambda)} \bigl( { \Vert {x - a} \Vert ^{2} + 2\mu \Vert x \Vert _{1} } \bigr), $$

*where* *μ* *is a certain positive number and* $\mathrm{S}_{\mu}$ *is the soft*-*thresholding operation defined by* $\mathrm{S}_{\mu}(a)_{i} = S_{\mu}(a_{i} )$, $i\in\Lambda$, *with*

$$S_{\mu}(z) = \textstyle\begin{cases} z - \mu,& z > \mu, \\ 0,& \vert z \vert \le\mu, \\ z + \mu,& z < - \mu. \end{cases} $$

For the $\ell_{1}$-ball $B_{R} = \{ {x \in\ell_{2} : \Vert x \Vert _{1} \le R} \}$ with $R: = \Vert {x^{\ast}} \Vert _{1} $, where $x^{\ast}\in\ell_{2} $ is the solution of problem (2.4), we replace the thresholding with the projection $\mathrm{P}_{B_{R} } $, and with a slight abuse of notation, we denote $\mathrm{P}_{B_{R} } $ by $\mathrm{P}_{R} $; then we introduce two properties of $\ell_{2}$-projections onto $\ell_{1}$-balls [25].

### Lemma 2.1

*For any* $a \in\ell_{2} $ *and for* $\mu> 0$, $\Vert {\mathrm{S}_{\mu}(a)} \Vert _{1} $ *is a piecewise linear*, *continuous*, *decreasing function of* *μ*; *moreover*, *if* $a \in\ell_{1}$, *then* $\Vert {\mathrm{S}_{0} (a)} \Vert _{1} = \Vert a \Vert _{1} $ *and* $\Vert {\mathrm{S}_{\mu}(a)} \Vert _{1} = 0$ *for* $\mu\ge\max_{i} \vert {a_{i} } \vert $.

### Lemma 2.2

*If* $\Vert a \Vert _{1} > R$, *then the* $\ell _{2}$ *projection of a on the* $\ell_{1}$-*ball with radius* *R* *is given by* $\mathrm{P}_{R} (a) = \mathrm{S}_{\mu}(a)$, *where* *μ* (*depending on* *a* *and* *R*) *is chosen such that* $\Vert {\mathrm{S}_{\mu}(a)} \Vert _{1} = R$. *If* $\Vert a \Vert _{1} \le R$, *then* $\mathrm{P}_{R} (a) = \mathrm{S}_{0} (a) = a$.

Next, we discuss a method to compute *μ*.

### Proposition 2.5

[25]

*For any* $a \in\Omega\subseteq\ell_{2}$, $\dim (\Omega) = n$, *sort the absolute values of the components of a by descending order*, *obtaining the rearranged sequence* $( {a_{i}^{\ast}} )_{i = 1,\ldots, n} $. *Then we perform a search to find* *k* *such that*

$$\bigl\Vert {\mathrm{S}_{a_{k}^{\ast}} (a)} \bigr\Vert _{1} = \sum _{i = 1}^{k - 1} { \bigl(a_{i}^{\ast}- a_{k}^{\ast}\bigr)} \le R < \sum_{i = 1}^{k} { \bigl(a_{i}^{\ast}- a_{k + 1}^{\ast}\bigr)} = \bigl\Vert {\mathrm{S}_{a_{k + 1}^{\ast}} (a)} \bigr\Vert _{1} $$

*or equivalently*,

$$\bigl\Vert {\mathrm{S}_{a_{k}^{\ast}} (a)} \bigr\Vert _{1} = \sum _{i = 1}^{k - 1} {i \bigl(a_{i}^{\ast}- a_{i + 1}^{\ast}\bigr)} \le R < \sum _{i = 1}^{k} {i \bigl(a_{i}^{\ast}- a_{i + 1}^{\ast}\bigr)} = \bigl\Vert {\mathrm{S}_{a_{k + 1}^{\ast}} (a)} \bigr\Vert _{1} . $$

*Set* $\nu: = k^{ - 1} ( {R - \Vert {\mathrm{S}_{a_{k}^{\ast}} (a)} \Vert _{1} } )$, *and* $\mu: = a_{k}^{\ast}+ \nu$, *then*

$$\begin{aligned} \bigl\Vert {{S_{\mu}}(a)} \bigr\Vert _{1} & = \sum_{i \in\Omega} {\max \bigl( \vert {{a_{i}}} \vert - \mu,0 \bigr)} = \sum_{i = 1}^{k} { \bigl(a_{i}^{*} - \mu \bigr)} \\ & = \sum_{i = 1}^{k - 1} { \bigl(a_{i}^{*} - a_{k}^{*} \bigr)} + k\nu = { \bigl\Vert {{S_{a_{k}^{*}}}(a)} \bigr\Vert _{1}} + k\nu = R. \end{aligned} $$

## CQ algorithms with two simpler variable stepsizes

In this section, two simpler variable stepsizes are proposed below. The advantages of the two stepsizes, comparing with (1.5) and (1.6), are that neither prior information about the matrix norm *A* nor any other conditions on *Q* and *A* are required.

### A simpler variable stepsize for CQ algorithm

We propose a new and simpler variable stepsize method for solving the feasibility problem. The algorithm is presented as follows.

#### Algorithm 3.1

*For any initial data* $x_{0} \in H_{1} $, $u \in H_{1} $ *and* $u \ne0$, *assume that the* *nth iterate* $x_{n} $ *has been constructed*; *then we compute the* $(n + 1)$ *th iterate* $x_{n + 1} $ *via the formula*

3.1 $$\begin{aligned}& \bar{x}_{n} = P_{C_{n} } \bigl( {t_{n} u + (1 - t_{n} )x_{n} } \bigr), \end{aligned}$$

3.2 $$\begin{aligned}& x_{n + 1} = P_{C_{n} } \bigl( {x_{n} - \tau_{n} \nabla f_{n} (x_{n} )} \bigr), \end{aligned}$$

*where*

3.3 $$ \tau_{n} : = \frac{\rho_{n} \Vert {x_{n} - \bar{x}_{n} } \Vert ^{2}}{ \Vert {Ax_{n} - A\bar{x}_{n} } \Vert ^{2}}, $$

*with* $\{ {\rho_{n} } \} \subset(0,2)$ *and* $\{t_{n} \} \subset(0,1)$. *If* $x_{n + 1} = x_{n} $ *or* $Ax_{n} = A\bar{x}_{n} $ *for some* $n \ge 0$, *then* $x_{n} $ *is a solution of SFP* (1.1) *and the iteration stops*; *otherwise*, *we set* $n: = n + 1$ *and go to* (3.1) *to compute the next iterate* $x_{n + 2} $.

#### Remark 3.1

We can easily approximate the upper bound *λ* of the eigenvalue interval to the symmetric matrix $A^{T}A$ from [1, 3], thus for any $x_{n \ge0} $, we can obtain ${\tau_{n}} \in (0,{2 / \lambda}) \subset(0,{2 / L})$, where *L* is the largest eigenvalue of $A^{T}A$.

Now we prove the convergence property of Algorithm 3.1.

#### Theorem 3.1

*If* $\Gamma\ne\emptyset$ *and* $\underline{\lim} _{n} {\tau_{n}}(2 - \lambda{\tau_{n}}) \ge\sigma > 0$, *the sequence* $\{x_{n} \}$ *generated by Algorithm* 3.1 *converges weakly to a solution of SFP* (1.1).

#### Proof

Let $x^{*} $ be a solution of SFP, since $C \subseteq C_{n} $, $Q \subseteq Q_{n} $, thus $x^{*} = P_{C} (x^{\ast}) = P_{C_{n} } (x^{\ast})$ and $Ax^{*} = P_{Q} (Ax^{\ast}) = P_{Q_{n} } (Ax^{\ast})$. It shows that $x^{\ast}\in C_{n} $ and $\nabla f_{n} (x^{\ast}) = 0$ for all $n = 0,1,\ldots$ , using (3.2) and ($\mathrm{p}_{4}$), we have

3.4 $$\begin{aligned} \bigl\Vert {x_{n + 1} - x^{*} } \bigr\Vert ^{2} =& \bigl\Vert {P_{C_{n} } \bigl(x_{n} - \tau_{n} \nabla f_{n} (x_{n} ) \bigr) - x^{*} } \bigr\Vert ^{2} \\ \le& \bigl\Vert { \bigl(x_{n} - x^{*} \bigr) - \tau_{n} \nabla f_{n} (x_{n} )} \bigr\Vert ^{2} \\ =& \bigl\Vert {x_{n} - x^{*} } \bigr\Vert ^{2} + \tau_{n}^{2} \bigl\Vert {\nabla f_{n} (x_{n} )} \bigr\Vert ^{2} - 2\tau_{n} \bigl\langle {x_{n} - x^{*} ,\nabla f_{n} (x_{n} )} \bigr\rangle \\ =& \bigl\Vert {x_{n} - x^{*} } \bigr\Vert ^{2} + \tau_{n}^{2} \bigl\Vert {\nabla f_{n} (x_{n} )} \bigr\Vert ^{2} \\ &{}- 2\tau_{n} \bigl\langle {x_{n} - x^{*} ,\nabla f_{n} (x_{n} ) - \nabla f_{n} \bigl(x^{\ast}\bigr)} \bigr\rangle \\ \le& \bigl\Vert {x_{n} - x^{*} } \bigr\Vert ^{2} + \tau _{n}^{2} L \bigl\Vert {(I - P_{Q_{n} } )Ax_{n} } \bigr\Vert ^{2} \\ &{} - 2\tau_{n} \bigl\langle {Ax_{n} - Ax^{*} ,(I - P_{Q_{n} } )Ax_{n} - (I - P_{Q_{n} } )Ax^{\ast}} \bigr\rangle \\ \le& \bigl\Vert {x_{n} - x^{*} } \bigr\Vert ^{2} + \tau _{n}^{2} L \bigl\Vert {(I - P_{Q_{n} } )Ax_{n} } \bigr\Vert ^{2} \\ &{}- 2\tau_{n} \bigl\Vert {(I - P_{Q_{n} } )Ax_{n} - (I - P_{Q_{n} } )Ax^{\ast}} \bigr\Vert ^{2} \\ =& \bigl\Vert {x_{n} - x^{*} } \bigr\Vert ^{2} + \bigl( \tau _{n}^{2} L - 2\tau_{n} \bigr) \bigl\Vert {(I - P_{Q_{n} } )Ax_{n} } \bigr\Vert ^{2}. \end{aligned}$$

Combining with Remark 3.1, we know $\tau_{n}^{2} L - 2\tau_{n} < 0$, thus it implies that the sequence $\{ { \Vert {x_{n} - x^{\ast}} \Vert ^{2}} \}$ is monotonically decreasing and hence $\{ {x_{n} } \} $ is bounded. Consequently, from (3.4) we get

3.5 $$ \lim_{n \to\infty} \bigl\Vert {(I - P_{Q_{n} } )Ax_{n} } \bigr\Vert ^{2} = 0. $$

Assume that *x̄* is an accumulation point of $\{x_{n} \}$ and $\{x_{n_{i} } \} \to\bar{x}$, $\{x_{n_{i} } \}$ is a subsequence of $\{x_{n} \}$. Then from (3.5) it follows

3.6 $$ \lim_{n_{i} \to\infty} \bigl\Vert {(I - P_{Q_{n_{i} } } )Ax_{n_{i} } } \bigr\Vert ^{2} = 0. $$

Next we show $\bar{x} \in\Gamma$.

Firstly, we show $\bar{x} \in C$. We prove it from two cases.

*Case 1*. $\lim_{n_{i} \to\infty} x_{n_{i} + 1} \ne\bar{x}$. Without loss of generality, we may assume $\lim_{n_{i} \to\infty} x_{n_{i} + 1} = \tilde{x} \ne\bar{x}$. Set $z_{n_{i} } = x_{n_{i} } - \tau_{n_{i} } \nabla f_{n_{i} } (x_{n_{i} } )$, so $x_{n_{i} + 1} = P_{C_{n_{i} } } (z_{n_{i} } )$ is the solution of the following programming:

$$\min\frac{1}{2} \Vert {z - z_{n_{i} } } \Vert ^{2} \quad \text{s.t. } c(x_{n_{i} } ) + \langle{\xi_{n_{i} } ,z - x_{n_{i} } } \rangle\le0. $$

By the Kuhn-Tucker condition, there exists a nonnegative number $\nu _{n_{i} } $ such that

3.7 $$\begin{aligned}& x_{n_{i} + 1} - z_{n_{i} } + \nu_{n_{i} } \xi_{n_{i} } = 0, \end{aligned}$$

3.8 $$\begin{aligned}& \nu_{n_{i} } \bigl(c(x_{n_{i} } ) + \langle{ \xi_{n_{i} } ,x_{n_{i} + 1} - x_{n_{i} } } \rangle \bigr) = 0. \end{aligned}$$

If there exist infinite $n_{i} $ such that $\nu_{n_{i} } = 0$ or $\xi _{n_{i} } = 0$, from (3.7) and (3.8) it leads to $\tilde{x} = \bar {x}$, so the contradiction happens. Therefore, $\nu_{n_{i} } > 0$ and $\xi_{n_{i} } \ne0$ for sufficiently large $n_{i} $. We go on to divide the discussion into two cases.

(1) If $\inf\{ \Vert {\xi_{n_{i} } } \Vert \} > 0$, we may assume $\xi _{n_{i} } \to\bar{\xi} \ne0$. Then $\bar{\xi} \in\partial c(\bar{x})$ by the lower semicontinuity of $\partial c(x)$. From (3.7) we have $\nu _{n_{i} } \to \langle{\bar{\xi},\tilde{x} - \bar{x}} \rangle/ \Vert \bar{\xi} \Vert ^{2} \stackrel{\Delta}{=} \bar {\nu}$. Thus we obtain from (3.7) and (3.8)

3.9 $$\begin{aligned}& \tilde{x} - \bar{x} + \bar{\nu}\bar{\xi} = 0, \end{aligned}$$

3.10 $$\begin{aligned}& \bar{\nu} \bigl(c(\bar{x}) + \langle{\bar{\xi},\tilde{x} - \bar{x}} \rangle \bigr) = 0. \end{aligned}$$

They mean that $\tilde{x} = P_{C(\bar{x})} (\bar{x})$.

Therefore, $\bar{x} \ne P_{C(\bar{x})} (\bar{x})$ by assumption. From ($\mathrm{p}_{5}$) we have

$$\bigl\Vert {\tilde{x} - x^{\ast}} \bigr\Vert ^{2} \le \bigl\Vert {\bar {x} - x^{\ast}} \bigr\Vert ^{2} - \Vert { \bar{x} - \tilde{x}} \Vert ^{2} < \bigl\Vert {\bar{x} - x^{\ast}} \bigr\Vert ^{2}. $$

Since $\{ \Vert {x_{n} - x^{\ast}} \Vert ^{2}\}$ is decreasing, both *x̄* and *x̃* are the accumulation points of $\{x_{n} \}$, then $\Vert {\tilde{x} - x^{\ast}} \Vert ^{2} = \Vert {\bar {x} - x^{\ast}} \Vert ^{2}$. It is a contradiction, so this case will not occur.

(2) If $\inf\{ \Vert {\xi_{n_{i} } } \Vert \} = 0$, then we get the lower semicontinuity of $\partial c(x)$. Since $c(x)$ is convex, then *x̄* is a minimizer of $c(x)$ over $H_{1} $. Since $c(x^{\ast}) \le0$, then $c(\bar{x}) \le c(x^{\ast}) \le0$. So $\bar{x} \in C$.

*Case 2*. $\lim_{n_{i} \to\infty} x_{n_{i} + 1} = \bar{x}$. As in Case 1, one has (3.7) and (3.8) by the Kuhn-Tucker condition. If there exist infinite $n_{i}$ s.t. $\nu_{n_{i} } = 0$ or $\xi_{n_{i} } = 0$, then we have $P_{C_{n_{i} } } (z_{n_{i} } ) = z_{n_{i} } $, so $c(x_{n_{i} } ) + \langle{\xi_{n_{i} } ,z_{n_{i} } - x_{n_{i} } } \rangle\le0$. Since

$$\lim_{n_{i} \to\infty} (z_{n_{i} } - x_{n_{i} } ) = \lim _{n_{i} \to\infty} \tau_{n_{i} } A^{T}(P_{Q_{n_{i} } } - I)Ax_{n_{i} } = 0, $$

then $c(\bar{x}) \le0$. Therefore, we have $\bar{x} \in C$.

Assume $\nu_{n_{i} } > 0$ and $\xi_{n_{i} } \ne0$ for sufficiently large $n_{i} $. If $\inf\{ \Vert {\xi_{n_{i} } } \Vert \} = 0$, such as above, it follows $\bar{x} \in C$. If $\inf\{ \Vert {\xi_{n_{i} } } \Vert \} > 0$, similar to Case 1(1), it leads to $\bar{x} = P_{C(\bar{x})} (\bar{x})$, which implies $c(\bar{x}) + \langle{\bar{\xi},\bar{x} - \bar{x}} \rangle\le0$. So $\bar{x} \in C$.

In summary, we can conclude $\bar{x} \in C$.

Secondly, we need to show $A\bar{x} \in Q$. From (3.5) we have

3.11 $$ \lim_{n_{i} \to\infty} \bigl\Vert {(I - P_{Q_{n_{i} } } )Ax_{n_{i} } } \bigr\Vert ^{2} = 0. $$

Since $P_{Q_{n_{i} } } (Ax_{n_{i} } ) \in Q_{n_{i} } $, we have

$$q(Ax_{n_{i} } ) + \bigl\langle {\eta_{n_{i} } ,P_{Q_{n_{i} } } (Ax_{n_{i} } ) - Ax_{n_{i} } } \bigr\rangle \le0. $$

Moreover, limiting the inequality and taking account of (3.11), we obtain that

$$q(A\bar{x}) \le0, $$

that is, $A\bar{x} \in Q$.

Therefore *x̄* is a solution of SFP. Thus we may replace $x^{*} $ in (3.4) with *x̄*, and get $\{ { \Vert {x_{n} - \bar{x}} \Vert } \}$ is convergent. Because there exists a subsequence $\{ { \Vert {x_{n_{i} } - \bar{x}} \Vert } \}$ convergent to 0, then $x_{n} \to \bar{x}$ as $n \to\infty$. □

### The other simpler variable stepsize for CQ algorithm

In this part, we introduce the other simpler choice of the stepsize $\tau_{n} $, which also is a variable stepsize to CQ algorithm. Either combining with the relaxed CQ algorithm [4], we have the next algorithm.

#### Algorithm 3.2

*Choose the initial data* $\forall x_{0} \in H_{1} $, *for* $u \in H_{1} $ *and* $u \ne 0$. *Assume that the* *nth iterate* $x_{n} $ *has been constructed*; *then we compute the* $(n + 1)$ *th iterate* $x_{n + 1} $ *via the formula*

3.12 $$\begin{aligned}& \bar{x}_{n} = P_{C_{n} } \bigl( {t_{n} u + (1 - t_{n} )x_{n} } \bigr), \end{aligned}$$

3.13 $$\begin{aligned}& x_{n + 1} = P_{C_{n} } \bigl( {x_{n} - \tau_{n} \nabla f_{n} (x_{n} )} \bigr), \end{aligned}$$

*where*

3.14 $$ \tau_{n} : = \frac{\rho_{n} \Vert {\bar{x}_{n} } \Vert ^{2}}{ \Vert {A\bar {x}_{n} } \Vert ^{2}}, $$

*with* $\{ {\rho_{n} } \} \subset(0,2)$ *and* $\{t_{n} \} \subset(0,1)$. *If* $x_{n + 1} = x_{n} $, *then stop and* $x_{n} $ *is a solution of SFP* (1.1); *otherwise*, *back to* (3.12) *and continue to compute* $x_{n + 2} $.

Obviously, (3.14) is also consistent with Remark 3.1. Thus, similar to the proof of Theorem 3.1, we can deduce that Algorithm 3.2 converges weakly to a solution of SFP (1.1).

## Two general KM-CQ algorithms with generalized variable stepsize

In this section, we integrate the variable stepsizes from (1.5) to (1.8) and obtain a variable stepsize that can cover them. After that, we apply it to improve the algorithms presented in [17] and [18] and construct two algorithms for approximating some solution of (1.1).

Let $\psi:C \to H_{1} $ be a *δ*-contraction with $\delta\in(0,1)$, let $r:H_{1} \to H_{1}\backslash\Theta$ and $q:{H_{1}} \to{H_{2}}\backslash \Theta$ be nonzero operators, where Θ denotes the zero point.

### A generalized variable stepsize for a general KM-CQ algorithm

The next recursion not only possesses a more generalized adaptive descent step, but it also can be implemented easily by the relaxed method.

#### Algorithm 4.1

*Choose the initial data* $x_{0} \in H_{1} $ *arbitrarily*. *Assume that the* *nth iterate* $x_{n} $ *has been constructed*; *then we compute the* $(n + 1)$ *th iterate* $x_{n + 1} $ *via the formula*

4.1 $$ x_{n + 1} = (1 - \beta_{n} )x_{n} + \beta_{n} P_{C_{n} } \bigl[ {\alpha_{n} \psi (x_{n} ) + (1 - \alpha_{n} )U_{n} x_{n} } \bigr], $$

*where* $U_{n} x_{n} = ( {I - \tau_{n} A^{T}(I - P_{Q_{n} } )A} )x_{n}$, $\tau_{n} : = \frac{\rho_{n} \Vert {r(x_{n} )} \Vert ^{2}}{ \Vert {Ar(x_{n} )} \Vert ^{2}}$ *or* ${\tau_{n}}: = \frac{{{\rho_{n}}{{ \Vert {p({x_{n}})} \Vert }^{2}}}}{{{{ \Vert {{A^{T}}p({x_{n}})} \Vert }^{2}}}}$, $\{ {\rho_{n} } \} \subset(0,2)$, $\{ t_{n} \} \subset(0,1)$, $\{ {\alpha_{n} } \}$ *and* $\{ {\beta_{n} } \}$ *are two real sequences in* $[0,1]$. *If* $x_{n + 1} = x_{n} $ *for some* $n \ge0$, *then* $x_{n} $ *is a solution of SFP* (1.1) *and the iteration stops*; *otherwise*, *continue to compute* $x_{n + 2} $.

#### Theorem 4.1

*Suppose that the SFP is consistent*, *that is*, $\Gamma= C \cap A^{ - 1}(Q) \ne\emptyset$, $\underline{\lim} _{n} {\tau_{n}}(2 - \lambda{\tau_{n}}) \ge\sigma > 0$. *Assume that the sequences* $\{ {\alpha_{n} } \}$ *and* $\{ {\beta_{n} } \}$ *satisfy the following conditions*:

($C_{1}$)
$\quad\lim_{n \to\infty} \alpha_{n} = 0$ *and* $\sum_{n = 1}^{\infty}{\alpha_{n} = \infty} $;
($C_{2}$)
$\quad0 < \underline{\lim} _{n} {\beta_{n}} $.

*Then* $\{ {x_{n} } \}$ *defined by* (4.1) *converges strongly to* $x^{\ast}= P_{\Gamma}\psi x^{\ast}$, *which solves the following variational inequality*:

4.2 $$ \bigl\langle { ( {\psi- I} )x^{\ast},y - x^{\ast}} \bigr\rangle \le0, \quad \forall y \in\Gamma. $$

#### Proof

Since $P_{\Gamma}:H_{1} \to\Gamma\subset C$ is nonexpansive and $\psi:C \to H_{1} $ is *δ*-contractive, therefore, we have $P_{\Gamma}\psi:C \to C$ is a contraction with $\delta\in(0,1)$. By the Banach contractive mapping principle, there exists a unique $x^{\ast}\in C$ such that $x^{\ast}= P_{\Gamma}\psi x^{\ast}$. By virtue of ($\mathrm{p}_{1}$), we see that (4.2) holds true.

By virtue of $x^{\ast}$ being a solution of the SFP, $x^{\ast}\in C \cap A^{ - 1}(Q)$, and $C \subseteq C_{n} $, $Q \subseteq Q_{n} $, then $x^{\ast}= P_{C} (x^{\ast}) = P_{C_{n} } (x^{\ast})$ and $Ax^{\ast}= P_{Q} (Ax^{\ast}) = P_{Q_{n} } (Ax^{\ast})$. From ($\mathrm{p}_{4}$) we have

4.3 $$\begin{aligned} \bigl\Vert {U_{n} x_{n} - x^{\ast}} \bigr\Vert ^{2} =& \bigl\Vert {x_{n} - \tau_{n} \nabla f_{n} (x_{n} ) - x^{\ast}} \bigr\Vert ^{2} \\ =& \bigl\Vert {x_{n} - x^{\ast}} \bigr\Vert ^{2} + \bigl\Vert {\tau_{n} \nabla f_{n} (x_{n} )} \bigr\Vert ^{2} - 2\tau_{n} \bigl\langle {x_{n} - x^{\ast},\nabla f_{n} (x_{n} )} \bigr\rangle \\ =& \bigl\Vert {x_{n} - x^{\ast}} \bigr\Vert ^{2} + \tau_{n}^{2} \bigl\Vert {\nabla f_{n} (x_{n} )} \bigr\Vert ^{2} \\ &{}- 2\tau_{n} \bigl\langle {Ax_{n} - Ax^{\ast},(I - P_{Q_{n} } )Ax_{n} - (I - P_{Q_{n} } )Ax^{\ast}} \bigr\rangle \\ \le& \bigl\Vert {x_{n} - x^{\ast}} \bigr\Vert ^{2} + \tau _{n}^{2} L \bigl\Vert {(I - P_{Q_{n} } )Ax_{n} } \bigr\Vert ^{2} \\ &{}- 2\tau_{n} \bigl\Vert {(I - P_{Q_{n} } )Ax_{n} } \bigr\Vert ^{2} \\ =& \bigl\Vert {x_{n} - x^{\ast}} \bigr\Vert ^{2} + \bigl( {\tau_{n}^{2} L - 2 \tau_{n} } \bigr) \bigl\Vert {(I - P_{Q_{n} } )Ax_{n} } \bigr\Vert ^{2}. \end{aligned}$$

Obviously, $\tau_{n} \in(0,2 / L)$ from Remark 3.1, then $\tau_{n}^{2} L - 2\tau_{n} < 0$, in particular, we obtain

4.4 $$ \bigl\Vert {U_{n} x_{n} - x^{\ast}} \bigr\Vert \le \bigl\Vert {x_{n} - x^{\ast}} \bigr\Vert . $$

At this point, we can establish the boundedness of $\{ {x_{n} } \}$. To see this, using (4.1) we have

$$\begin{aligned}& \bigl\Vert {x_{n + 1} - x^{\ast}} \bigr\Vert \\& \quad = \bigl\Vert {(1 - \beta_{n} )x_{n} + \beta_{n} P_{C_{n} } \bigl[ {\alpha_{n} \psi(x_{n} ) + (1 - \alpha_{n} )U_{n} x_{n} } \bigr] - x^{\ast}} \bigr\Vert \\& \quad \le (1 - \beta_{n} ) \bigl\Vert {x_{n} - x^{\ast}} \bigr\Vert + \beta_{n} \bigl\Vert {P_{C_{n} } \bigl[ {\alpha_{n} \psi(x_{n} ) + (1 - \alpha_{n} )U_{n} x_{n} } \bigr] - x^{\ast}} \bigr\Vert \\& \quad \le (1 - \beta_{n} ) \bigl\Vert {x_{n} - x^{\ast}} \bigr\Vert + \beta_{n} \bigl\Vert { \alpha_{n} \psi(x_{n} ) + (1 - \alpha_{n} )U_{n} x_{n} - x^{\ast}} \bigr\Vert \\& \quad = (1 - \beta_{n} ) \bigl\Vert {x_{n} - x^{\ast}} \bigr\Vert \\& \quad\quad{} + \beta_{n} \bigl\Vert {\alpha_{n} \bigl( { \psi(x_{n} ) - \psi \bigl(x^{\ast}\bigr)} \bigr) + (1 - \alpha_{n} ) \bigl( {U_{n} x_{n} - x^{\ast}} \bigr) + \alpha_{n} \psi \bigl(x^{\ast}\bigr) - \alpha_{n} x^{\ast}} \bigr\Vert \\& \quad \le (1 - \beta_{n} ) \bigl\Vert {x_{n} - x^{\ast}} \bigr\Vert + \alpha_{n} \beta_{n} \bigl\Vert {\psi(x_{n} ) - \psi \bigl(x^{\ast}\bigr)} \bigr\Vert + (1 - \alpha_{n} )\beta_{n} \bigl\Vert {U_{n} x_{n} - x^{\ast}} \bigr\Vert \\& \quad\quad{} + \alpha_{n} \beta _{n} \bigl\Vert {\psi \bigl(x^{\ast}\bigr) - x^{\ast}} \bigr\Vert \\& \quad \le \bigl( {1 - (1 - \delta)\alpha_{n} \beta_{n} } \bigr) \bigl\Vert {x_{n} - x^{\ast}} \bigr\Vert + \alpha_{n} \beta_{n} \bigl\Vert {\psi \bigl(x^{\ast}\bigr) - x^{\ast}} \bigr\Vert \\& \quad = \bigl( {1 - (1 - \delta)\alpha_{n} \beta_{n} } \bigr) \bigl\Vert {x_{n} - x^{\ast}} \bigr\Vert + (1 - \delta) \alpha _{n} \beta_{n} \frac{ \Vert {\psi(x^{\ast}) - x^{\ast}} \Vert }{1 - \delta} \\& \quad \le \max \biggl\{ { \bigl\Vert {x_{0} - x^{\ast}} \bigr\Vert ,\frac{ \Vert {\psi(x^{\ast}) - x^{\ast}} \Vert }{1 - \delta}} \biggr\} = M, \end{aligned}$$

for all $n \ge0$, which indicates $\{ {x_{n} } \}$ is bounded. Set ${z_{n}} = {P_{{C_{n}}}} [ {{\alpha_{n}}\psi({x_{n}}) + (1 - {\alpha_{n}}){U_{n}}{x_{n}}} ]$, thus $\{ {z_{n}}\} $ is also bounded.

Next, we prove $x_{n} \to x^{\ast}$ ($n \to\infty$). By virtue of (4.1), Proposition 2.1($\mathrm{p}_{2}$) and (4.4), we have

4.5 $$ \begin{aligned}[b] & \bigl\Vert {x_{n + 1} - x^{\ast}} \bigr\Vert ^{2} \\ &\quad = \bigl\Vert {(1 - \beta_{n} )x_{n} + \beta_{n} P_{C_{n} } \bigl[ {\alpha_{n} \psi (x_{n} ) + (1 - \alpha_{n} )U_{n} x_{n} } \bigr] - x^{\ast}} \bigr\Vert ^{2} \\ &\quad \le(1 - \beta_{n} ) \bigl\Vert {x_{n} - x^{\ast}} \bigr\Vert ^{2} + \beta_{n} \bigl\Vert {P_{C_{n} } \bigl[ {\alpha_{n} \psi(x_{n} ) + (1 - \alpha_{n} )U_{n} x_{n} } \bigr] - x^{\ast}} \bigr\Vert ^{2}, \end{aligned} $$

where we set

$$\begin{aligned}& \bigl\Vert {P_{C_{n} } \bigl[ {\alpha_{n} \psi(x_{n} ) + (1 - \alpha_{n} )U_{n} x_{n} } \bigr] - x^{\ast}} \bigr\Vert ^{2} \\& \quad = \bigl\Vert {P_{C_{n} } [ {w_{n} } ] - x^{\ast}} \bigr\Vert ^{2} \\& \quad = \bigl\langle {P_{C_{n} } [ {w_{n} } ] - x^{\ast},P_{C_{n} } [ {w_{n} } ] - x^{\ast}} \bigr\rangle \\& \quad = \bigl\langle {P_{C_{n} } [ {w_{n} } ] - w_{n} ,P_{C_{n} } [ {w_{n} } ] - x^{\ast}} \bigr\rangle + \bigl\langle {w_{n} - x^{\ast},P_{C_{n} } [ {w_{n} } ] - x^{\ast}} \bigr\rangle , \end{aligned}$$

since $\langle{P_{C_{n} } [ {w_{n} } ] - w_{n} ,P_{C_{n} } [ {w_{n} } ] - x^{\ast}} \rangle\le0$, we have

$$\begin{aligned}& \bigl\Vert {P_{C_{n} } [ {w_{n} } ] - x^{\ast}} \bigr\Vert ^{2} \\& \quad \le \bigl\langle {w_{n} - x^{\ast},P_{C_{n} } [ {w_{n} } ] - x^{\ast}} \bigr\rangle \\& \quad = \bigl\langle {\alpha_{n} \psi(x_{n} ) + (1 - \alpha_{n} )U_{n} x_{n} - x^{\ast},P_{C_{n} } [ {w_{n} } ] - x^{\ast}} \bigr\rangle \\& \quad = \bigl\langle {\alpha_{n} \bigl( {\psi(x_{n} ) - \psi \bigl(x^{\ast}\bigr)} \bigr) + (1 - \alpha_{n} ) \bigl( {U_{n} x_{n} - x^{\ast}} \bigr)+ \alpha_{n} \bigl( {\psi \bigl(x^{\ast}\bigr) - x^{\ast}} \bigr),P_{C_{n} } [ {w_{n} } ] - x^{\ast}} \bigr\rangle \\& \quad \le \bigl( {\alpha_{n} \bigl\Vert {\psi(x_{n} ) - \psi \bigl(x^{\ast}\bigr)} \bigr\Vert + (1 - \alpha_{n} ) \bigl\Vert {U_{n} x_{n} - x^{\ast}} \bigr\Vert } \bigr) \bigl\Vert {P_{C_{n} } [ {w_{n} } ] - x^{\ast}} \bigr\Vert \\& \quad\quad{} + \alpha_{n} \bigl\langle {\psi \bigl(x^{\ast}\bigr) - x^{\ast},P_{C_{n} } [ {w_{n} } ] - x^{\ast}} \bigr\rangle \\& \quad \le \bigl( {1 - ( {1 - \delta} )\alpha_{n} } \bigr) \bigl\Vert {x_{n} - x^{\ast}} \bigr\Vert \bigl\Vert {P_{C_{n} } [ {w_{n} } ] - x^{\ast}} \bigr\Vert + \alpha_{n} \bigl\langle {\psi \bigl(x^{\ast}\bigr) - x^{\ast},P_{C_{n} } [ {w_{n} } ] - x^{\ast}} \bigr\rangle \\& \quad \le \frac{1 - ( {1 - \delta} )\alpha_{n} }{2} \bigl\Vert {x_{n} - x^{\ast}} \bigr\Vert ^{2} + \frac{1}{2} \bigl\Vert {P_{C_{n} } [ {w_{n} } ] - x^{\ast}} \bigr\Vert ^{2} \\& \quad \quad {} + \alpha_{n} \bigl\langle {\psi \bigl(x^{\ast}\bigr) - x^{\ast},P_{C_{n} } [ {w_{n} } ] - x^{\ast}} \bigr\rangle . \end{aligned}$$

Therefore,

4.6 $$ \bigl\Vert {P_{C_{n} } [ {w_{n} } ] - x^{\ast}} \bigr\Vert ^{2} \le \bigl( {1 - ( {1 - \delta} ) \alpha_{n} } \bigr) \bigl\Vert {x_{n} - x^{\ast}} \bigr\Vert ^{2} + 2\alpha_{n} \bigl\langle {\psi \bigl(x^{\ast}\bigr) - x^{\ast},P_{C_{n} } [ {w_{n} } ] - x^{\ast}} \bigr\rangle . $$

Substituting (4.6) into (4.5) can yield

4.7 $$ \begin{aligned}[b] & \bigl\Vert {x_{n + 1} - x^{\ast}} \bigr\Vert ^{2} \\ &\quad \le(1 - \beta_{n} ) \bigl\Vert {x_{n} - x^{\ast}} \bigr\Vert ^{2} \\ &\quad \quad{}+ \bigl( {1 - ( {1 - \delta} )\alpha_{n} } \bigr) \beta_{n} \bigl\Vert {x_{n} - x^{\ast}} \bigr\Vert ^{2} + 2\alpha_{n} \beta_{n} \bigl\langle { \psi \bigl(x^{\ast}\bigr) - x^{\ast},P_{C_{n} } [ {w_{n} } ] - x^{\ast}} \bigr\rangle \\ &\quad \le \bigl( {1 - ( {1 - \delta} )\alpha_{n} \beta_{n} } \bigr) \bigl\Vert {x_{n} - x^{\ast}} \bigr\Vert ^{2} + ( {1 - \delta} )\alpha_{n} \beta_{n} \frac{2}{1 - \delta} \bigl\langle {\psi \bigl(x^{\ast}\bigr) - x^{\ast},P_{C_{n} } [ {w_{n} } ] - x^{\ast}} \bigr\rangle . \end{aligned} $$

Since $x^{\ast}\in C \subseteq C_{n} $, $P_{C_{n} } :H_{1} \to C \subseteq C_{n} $ and $\psi:C \subseteq C_{n} \to H_{1} $, then $P_{C_{n} } \psi:C_{n} \to C_{n} $, $x^{\ast}= P_{C_{n} } \psi x^{\ast}$.

Due to the property of the projection ($\mathrm{p}_{1}$) in Proposition 2.1,

4.8 $$ \begin{aligned}[b] &\limsup_{n \to\infty} \bigl\langle {\psi \bigl(x^{\ast}\bigr) - x^{\ast},P_{C_{n} } [ {w_{n} } ] - x^{\ast}} \bigr\rangle \\ &\quad = \max_{P_{C_{n} } [ {w_{n} } ] \in C_{n} } \bigl\langle {\psi \bigl(x^{\ast}\bigr) - P_{C_{n} } \psi \bigl(x^{\ast}\bigr),P_{C_{n} } [ {w_{n} } ] - P_{C_{n} } \psi \bigl(x^{\ast}\bigr)} \bigr\rangle \le0. \end{aligned} $$

Applying (4.8) and Proposition 2.3 to (4.7), we deduce that $x_{n} \to x^{\ast}$.

Assume that *x̂* is an accumulation point of $\{ {x_{n} } \}$ and $x_{n_{i} } \to\hat{x}$, where $\{x_{n_{i} } \}_{i = 1}^{\infty}$ is a subsequence of $\{ {x_{n} } \}$. Next we will prove that *x̂* is a solution of SFP.

As $x_{n} \to x^{\ast}$, we know $x_{n} \rightharpoonup x^{\ast}$, that is,

4.9 $$ \lim_{n \to\infty} \Vert {x_{n + 1} - x_{n} } \Vert = 0. $$

Therefore, limit (4.4), we can obtain

$$\lim_{n \to\infty} \bigl\Vert { U_{n}(x_{n})-x^{\ast}} \bigr\Vert = 0. $$

Then, limit (4.3), we get

4.10 $$ \lim_{n \to\infty} \bigl\Vert {(I - P_{Q_{n} } )Ax_{n} } \bigr\Vert = 0. $$

On the one hand, we show $\hat{x} \in C$.

Notice that $x_{n_{i} } \to\hat{x}$ and $x_{n_{i + 1} } - x_{n_{i} } \to 0$ ($i \to\infty$). Since $x_{n_{i + 1} } \in C_{n_{i} } $, then by virtue of the definition of $C_{n_{i} } $, we have

$$c(x_{n_{i} } ) + \langle{\xi_{n_{i} } ,x_{n_{i + 1} } - x_{n_{i} } } \rangle\le0, \quad \forall i = 1,2,\ldots, $$

taking the limit and using (4.9), we obtain that

$$c(\hat{x}) \le0. $$

Hence, we get $\hat{x} \in C$.

On the other hand, we need to show $A\hat{x} \in Q$.

Since $P_{Q_{n_{i} } } (Ax_{n_{i} } ) \in Q_{n_{i} } $, we have

$$q(Ax_{n_{i} } ) + \bigl\langle {\eta_{n_{i} } ,P_{Q_{n_{i} } } (Ax_{n_{i} } ) - Ax_{n_{i} } } \bigr\rangle \le0, $$

taking $n_{i} \to\infty$, by virtue of (4.10), we deduce that

$$q(A\hat{x}) \le0, $$

that is, $A\hat{x} \in Q$.

Therefore, *x̂* is a solution of SFP.

Thus we may replace $x^{*} $ in (4.7) with *x̂* and get $\{ { \Vert {x_{n} - \hat{x}} \Vert } \}$ is convergent. Because there exists a subsequence $\{ { \Vert {x_{n_{i} } - \hat{x}} \Vert } \}$ convergent to 0, then $x_{n} \to\hat{x}$ as $n \to\infty$. □

### The other extended algorithm

Let $h:C \to H_{1} $ be a *κ*-contraction. Let $B:H_{1} \to H_{1} $ be a self-adjoint strongly positive bounded linear operator with coefficient $\lambda\in(0,1)$, for $\forall x \in H_{1} $, there exists $\langle{Bx,x} \rangle\ge\lambda \Vert x \Vert ^{2}$. Take a constant *σ* such that $0 < \sigma\kappa< \lambda$.

As *B* is self-adjoint, we have$\Vert B \Vert = \sup_{ \Vert x \Vert = 1} \langle{Bx,x} \rangle$. $I - B$ is also self-adjoint, then

$$\begin{aligned} \Vert {I - B} \Vert =& \sup_{ \Vert x \Vert = 1} \bigl\langle {(I - B)x,x} \bigr\rangle = \sup_{ \Vert x \Vert = 1} \bigl\{ { \Vert x \Vert ^{2} - \langle{Bx,x} \rangle} \bigr\} \\ \le& \sup_{ \Vert x \Vert = 1} \bigl\{ { ( {1 - \lambda} ) \Vert x \Vert ^{2}} \bigr\} \le 1 - \lambda. \end{aligned}$$

In (4.1), we set $\psi(x) = \sigma h(x) + (I - B)U_{n} x$, thus

$$\begin{aligned} \bigl\Vert {\psi(x) - \psi(y)} \bigr\Vert \le& \sigma\kappa \Vert {x - y} \Vert + \Vert {I - B} \Vert \Vert {x - y} \Vert \\ \le& (\sigma\kappa+ 1 - \lambda ) \Vert {x - y} \Vert , \end{aligned}$$

for $\forall x,y \in H_{1} $, we know that $\sigma\kappa+ 1 - \lambda \in(0,1)$, $\psi:C \to H_{1} $ is still a contraction. Accordingly, we have the following extended algorithm that is a special case of Algorithm 4.1.

#### Algorithm 4.2

*Choose the initial data* $x_{0} \in H_{1} $ *arbitrarily*. *Assume that the* *nth iterate* $x_{n} $ *has been constructed*; *then we compute the* $(n + 1)$ *th iterate* $x_{n + 1} $ *via the formula*

4.11 $$ x_{n + 1} = (1 - \beta_{n} )x_{n} + \beta_{n} P_{C_{n} } \bigl[ {\alpha_{n} \sigma h(x_{n} ) + (I - \alpha_{n} B)U_{n} x_{n} } \bigr], $$

*where* $U_{n} x_{n} = ( {I - \tau_{n} A^{T}(I - P_{Q_{n} } )A} )x_{n} $, $\tau_{n} : = \frac{\rho_{n} \Vert {r(x_{n} )} \Vert ^{2}}{ \Vert {Ar(x_{n} )} \Vert ^{2}}$ *or* ${\tau_{n}}: = \frac{{{\rho_{n}}{{ \Vert {p({x_{n}})} \Vert }^{2}}}}{{{{ \Vert {{A^{T}}p({x_{n}})} \Vert }^{2}}}}$, $\tau_{n} \in(0,2 / L)$, $\{ {\rho_{n} } \} \subset (0,2)$, $\{t_{n} \} \subset(0,1)$, $\{ {\alpha_{n} } \}$ *and* $\{ {\beta_{n} } \}$ *are two real sequences in* $[0,1]$. *If* $x_{n + 1} = x_{n} $, *then stop and* $x_{n} $ *is a solution of SFP* (1.1); *otherwise*, *continue to compute* $x_{n + 2} $.

#### Theorem 4.2

*Suppose that the SFP is consistent*, *that is*, $\Gamma=C \cap A^{ - 1}(Q) \ne\emptyset$, $\underline{\lim} _{n} {\tau_{n}}(2 - \lambda{\tau_{n}}) \ge\sigma > 0$, *assume that the sequences* $\{ {\alpha_{n} } \} $ *and* $\{ {\beta_{n} } \}$ *satisfy the following conditions*:

($C_{1}$)
$\lim_{n \to\infty} \alpha_{n} = 0$ *and* $\sum_{n = 1}^{\infty}{\alpha_{n} = \infty} $;
($C_{2}$)
$0 < \underline{\lim} _{n} {\beta_{n}} $.

*Then* $\{ {x_{n} } \}$ *defined by* (4.11) *converges strongly to* $x^{\ast}= P_{\Gamma}[\sigma h(x^{\ast})+(I-B)U_{n} x^{\ast}] $, *which solves the following variational inequality*:

$$\bigl\langle {\sigma h \bigl(x^{\ast}\bigr) - B \bigl(x^{\ast}\bigr),y - x^{\ast}} \bigr\rangle \le 0, \quad \forall y \in\Gamma. $$

## Numerical experiments and results

This section considers two numerical experiments to illustrate the performance of the above proposed variable stepsizes in CQ algorithm. Firstly, we see that a great amount of problems in signal and image processing can be seen as estimating $x \in\mathrm{R}^{N}$ from the linear observation model

5.1 $$ y = Ax + \varepsilon, $$

where $y \in\mathrm{R}^{M}$ is the observed or measured data with noisy *ε*. $A:\mathrm{R}^{N} \to\mathrm{R}^{M}$ denotes the bounded linear observation or measurement operator. Sometimes, the range of *A* may not be closed in most inverse problems, therefore, if *A* is ill-conditioned, the problem will be ill-posed.

If *x* is a sparse expansion, finding the solutions of (5.1) can be seen as finding a solution to the least-square problem

5.2 $$ \min_{x \in\mathrm{R}^{N}} \frac{1}{2} \Vert {y - Ax} \Vert ^{2} \quad\text{subject to } \Vert x \Vert _{1} < t $$

for any real number $t > 0$.

Problem (5.2) is a particular case of SFP (1.1) where $C = \{ {x \in\mathrm{R}^{N}: \Vert x \Vert _{1} \le t} \}$ and $Q = \{y\}$, i.e., find $\Vert x \Vert _{1} \le t$ such that $Ax=y$. Therefore, CQ algorithm can be applied to solve (5.2). From Propositions 2.4 and 2.5 the projection onto *C* can be easily computed [15], while Lemmas 2.1 and 2.2 show the special situation of Proposition 2.4.

Next, following the experiments in [15, 26], we choose two particular problems, i.e., the compressed sensing and the image deconvolution, which are covered by (5.1). The experiments compare the performances of the proposed stepsizes of the CQ algorithm in this paper with the stepsizes in [15] and [16].

### Compressed sensing

**Figure 1:**
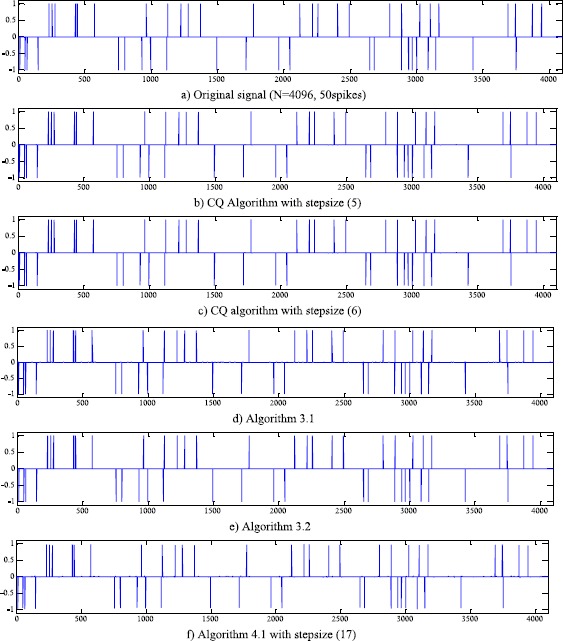
**Compressed sensing problem, from top to bottom: original signal, results of CQ algorithm with stepsizes (** **1.5** **), (** **1.6** **), (** **3.3** **) and (** **3.14** **), the last is KM-CQ algorithm with stepsize (** **3.3** **).**

We consider a typical compressed sensing model, where a sparse signal recovery problem with a signal $x \in\mathrm{R}^{N}$, and $N = 2^{12}$. This original signal *x* contains only $m=50$ spikes with amplitude ±1, and the spikes are located at random, see the top of Figure 1. *x* is being reconstructed from $M = 2^{10}$ measurements, thus *A* is a $M\times N$ matrix randomly obtained with independent samples of a orthonormalized standard Gaussian distribution, and the noisy *ε* is with variance $\sigma_{\varepsilon}^{2} = 10^{ - 4}$. To (5.2), we also set *t*=50.

For stepsizes (1.5) and (1.6) in [15] and [16], respectively, we still consider the stepsize with constant $\rho= 2$, $\omega_{n} = (n + 2)^{ - 5}$, while for stepsizes (3.3) and (3.14), we set $\rho = 1$. For (3.1) and (3.12), we set $t = 0.1$ and $u = \operatorname{rand} ( {N,1} ) \in(0,1)$. For (4.1) we use (3.1) and (3.3), and set $\alpha _{n} = (n + 2)^{ - 1}$, $\beta _{n} = 1 - (n + 2)^{ - 1}$ and $\psi\equiv0$ as its special case. All the processes are started with the initial signal $x_{0} = 0$ and finished with the stop rule

$${ \Vert {x_{n + 1} - x_{n} } \Vert } / { \Vert {x_{n} } \Vert } < 10^{ - 3}. $$

We also calculated the mean squared error (MSE) for the results

$$\mathit{MSE} = ( {1 / N} ) \bigl\Vert {x^{\ast}- x} \bigr\Vert , $$

where $x^{\ast}$ is an estimated signal of *x*.

The simulated results of different algorithms with different steps can be seen in Figure 1 and Table 1. Algorithms 3.1 and 3.2 have less iteration steps and smaller MSE, especially for Algorithm 3.1. Thus, we see that stepsizes (3.3) and (3.14) not only have simper formats than before, but also can make CQ algorithms have faster iteration and better restored precision.

**Table 1:** **Results of different stepsizes and algorithms for Figure** **1**

**Algorithms**	**MSE**	***n***	**CPU time (s)**
CQ with (1.5)	2.8021e−005	66	0.8504
CQ with (1.6)	2.844e−005	66	0.8579
3.1	5.1714e−006	25	0.6858
3.2	1.5253e−006	64	1.5685

### Image deconvolution

In this subsection, we apply the CQ algorithms in the paper to image deconvolution. The observation model can also be described as (5.1), we wish to estimate an original image *x* from an observation *y*, while matrix *A* represents the observation operator, and *ε* is a sample of a zero-mean white Gaussian field of variance *σ*. For the 2D image deconvolution problem, *A* is a block-circulant matrix with circulant blocks [27]. We stress that the goal of these experiments is not to assess the restored precision of the algorithms, but to apply the algorithms in paper to solve this particular SFP, then compare the iterative speed and restored precision of the proposed stepsizes against the CQ algorithms.

According to papers [26, 27], we also take the well-known Cameraman image. In the experiments, we employ Haar wavelets, and the blur point spread functions are uniform blur with size $9\times9$, $h_{ij} = (1 + i^{2} + j^{2})^{ - 1}$, for $i,j = - 4,\ldots,4$ and for $i,j = - 7,\ldots,7$. The noise variance is $\sigma^{2} = 0.308$, 2 and 8, respectively. We have $N = M = 256^{2}$, then the block-circulant matrix *A* can be constructed by the blur point spread functions, and *A* may be very ill-conditioned. Set all the threshold values $\mu= 0.25$, *t* is the sum of all the pixel values in the original image. Moreover, we use $y=\mathit{IFFT}(\mathit{FFT}(A).*\mathit{FFT}(x))+\varepsilon$ to obtain the observation, where *FFT* is the fast Fourier transform, *IFFT* is the inverse fast Fourier transform. Other settings in the above stepsizes and algorithms are the same as in 4.1. We set the initial image $x_{0} = 0$ and also follow the stop rule ${ \Vert {x_{n + 1} - x_{n} } \Vert } / { \Vert {x_{n} } \Vert } < 10^{ - 3}$.

The results of iteration steps, CPU time and the SNR improvements are presented in Table 2. It also testifies that the proposed stepsizes and algorithms in this paper can give better performance.

**Table 2:** **Results for the restorations of different stepsizes and algorithms**

**Blur kernel**	$\boldsymbol{\sigma^{2}}$	**Algorithms**	**SNR (dB)**	***n***	**CPU time (s)**
9 × 9 uniform	0.308	CQ with (1.5)	16.1802	52	3.3883
		CQ with (1.6)	16.1722	48	3.1636
		3.1	14.5266	19	1.3847
		3.2	14.5265	19	1.3199
		4.1 with (3.3)	14.2464	34	2.4280
$h_{ij}=(1+i^{2}+j^{2})^{ - 1}$ for *i*,*j* = −4,…,4	2	CQ with (1.6)	22.6184	19	1.3007
		CQ with (1.5)	22.4329	17	1.1718
		3.1	19.8401	14	1.0746
		3.2	19.8401	14	1.0266
		4.1 with (3.3)	19.4912	33	2.3306
$h_{ij}=(1+i^{2}+j^{2})^{ - 1}$ for *i*,*j* = −7,…,7	8	CQ with (1.6)	12.7305	39	2.6739
		CQ with (1.5)	14.6363	42	2.8604
		3.1	19.3561	18	1.3390
		3.2	19.3560	18	1.3057
		4.1 with (3.3)	18.6140	33	2.3628

## Conclusions and discussion

In this paper, we have proposed two simpler variable stepsizes for the CQ algorithm. Compared with the other related variable stepsizes, they also need not to compute the largest eigenvalue of *A* and can be calculated easily. Furthermore, we also presented a more general KM-CQ algorithm with generalized variable stepsizes. As a special case, we deduced another general format. Obviously, both the general algorithms with the generalized variable stepsizes can solve the SFP and some special variational inequality problem better. The corresponding weak and strong convergence properties have also been established. In the experiments, through the compressed sensing and image deconvolution models, we compare the proposed stepsizes with the former ones, the results obtained from the proposed stepsizes and algorithms appear to be significantly better.

We should notice that the values of parameter $\rho_{n} $ are fixed in the above experiments. Actually, a different value of $\rho_{n} $ can also affect the convergence speed of the algorithms. Therefore, our future work is to find the method to choose a self-adaptive sequence $\{ {\rho_{n} } \}$.
